# Optimizing Selective RF Pulses for Enhanced Signal Stability in Turbo Spin Echo Using a Differentiable Extended Phase Graph Model

**DOI:** 10.1002/mrm.70340

**Published:** 2026-03-12

**Authors:** Madison M. Augelli, Anuj Sharma, Mark A. Griswold, William A. Grissom

**Affiliations:** ^1^ Biomedical Engineering Case Western Reserve University Cleveland Ohio USA; ^2^ Department of Radiology Case Western Reserve University Cleveland Ohio USA

**Keywords:** extended phase graph, optimization, RF pulse design, turbo spin echo

## Abstract

**Purpose:**

To improve slice profile consistency across echo trains in turbo spin echo (TSE) imaging, thereby reducing image blurring and increasing the accuracy of multi echo spin echo $T_{2}$ mapping.

**Methods:**

Excitation and refocusing RF pulses were optimized for TSE using a differentiable extended phase graph model that incorporates the spinor profiles of the RF pulses to calculate the magnetization slice profile across the echo train. The pulses were optimized using an L‐BFGS algorithm in PyTorch to minimize an error term on the target signal magnitude with singular value regularization to promote similarity. The performance of the optimized pulses was assessed by comparing to time bandwidth‐matched SLR RF pulses. Slice profile consistency was calculated in simulation and in a homogeneous phantom. Images were acquired in vivo to assess blurring artifacts, and $T_{2}$ measurements were acquired in a NIST phantom and in vivo to assess improvements in accuracy.

**Results:**

The optimized pulses demonstrated superior performance over time bandwidth‐matched SLR pulses, with a 90% reduction in the standard deviation of the normalized integrated signal at each echo. Optimized pulses increased sharpness in vivo at the edges of CSF and veins perpendicular to the phase‐encoded direction, reduced $T_{2}$ mapping error in the NIST phantom by 91%, and produced more accurate in vivo $T_{2}$ maps.

**Conclusion:**

The optimization method enables flexible design of RF pulses in echo train pulse sequences with consistent slice profiles, achieving a target signal progression while also maintaining a constant phase and FWHM between echoes.

## 1 Introduction

Turbo spin echo (TSE) imaging is a workhorse of modern MRI [1]. In 2D TSE, a slice‐selective RF excitation pulse is followed by a series of $N_{\text{ETL}}$ (echo train length) slice‐selective refocusing pulses surrounded by crusher gradients, with a different line of k‐space acquired after each refocusing pulse, allowing for $N_{\text{ETL}}$ lines of k‐space to be collected each TR. This makes TSE a fast‐imaging sequence that provides high resolution, flexible contrast, and minimal artifacts. However, TSE suffers from blurring mainly due to two sources that cause signal differences in echoes. The first and unavoidable source is relaxation of the transverse magnetization between echoes, and efforts to decrease blurring have principally focused on this source. These efforts include optimizing the flip angle train to balance transverse and longitudinal signal decay [2, 3, 4, 5, 6], filtering prior to reconstruction based on the estimated $T_{2}$ value of the sample [7], and the use of convolutional neural networks to improve the sharpness of TSE images after reconstruction [5].

The second source of blurring in 2D TSE is differences in the signal profile in the slice dimension. At 3T, 2D TSE sequences generally use sub‐180° refocusing pulses to limit RF energy deposition and maintain high echo signal amplitudes [8]. The same pulse shape is generally used for each refocusing pulse by scaling it according to flip angle; however, due to the nonlinearity of the Bloch equations at high flip angles and evolving magnetization pathways, the slice profiles excited by these scaled pulses vary nonlinearly, particularly in the transition bands. Thus, a slightly different slice profile shape is measured at each line of k‐space, and because these shapes vary smoothly over echoes, the variations result in blurring in the images. They also lead to the sampling of a slightly different volumes of tissue at each echo.

Selective RF pulse design in MRI typically assumes that the initial magnetization is in a uniform state. The assumption does not hold for refocusing RF pulses in TSE because the preceding pulses generate varying mixtures of spin and stimulated echoes, altering the magnetization state entering each refocusing pulse. Although SLR [9] and optimal control [10] allow the user to specify arbitrary target slice profiles, most implementations of these algorithms still assume an ideal initial magnetization state when defining a target rotation or magnetization pattern. Additionally, scaling the refocusing pulses designed by these methods does not linearly scale the resulting slice profile because of the nonlinearity of the Bloch equations at high flip angles. Thus, these approaches alone cannot fully mitigate slice profile inconsistency across echoes in 2D TSE sequences. Deep learning approaches such as physics‐inspired neural networks have been used to design RF pulses to match desired slice profiles [11, 12], but these also do not consider the impact of stimulated echoes on the magnetization. The only previous approach to directly optimize RF pulse shapes for signal slice profile consistency, in consideration of previous RF pulses uses recursive algebra to adapt the SLR algorithm to produce RF pulses with consistent slice profiles across two or three shots in GRE VFA FLEET sequences [13]. That methodology, however, was specific to the GRE VFA FLEET pulse sequence, and does not generalize.

We propose to jointly design all the selective RF pulses in a TSE echo train based on a comprehensive model of the magnetization pathways evolving between echoes, to enhance signal stability by generating consistent signal profiles across the echoes. The method is based on a differentiable partitioned extended phase graph (EPG) forward model that tracks the magnetization profile across locations within the imaged slice at each echo and accommodates shaped RF pulses [14, 15]. Its differentiability enables the pulse shapes to be jointly optimized to control the shapes of the signal profiles at the echoes and promote similarity, thereby decreasing 2D TSE image blurring and improving multi‐echo spin echo (MESE) $T_{2}$ mapping accuracy. In the following sections the EPG‐based forward model of the evolving magnetization pathways in TSE and the RF pulse optimization strategy are described. Simulation, phantom, and in vivo experiments are then reported, which compare the slice profiles, in vivo blurring, and $T_{2}$ mapping of the optimized pulses with parameter‐matched SLR pulses.

## 2 Theory

### 2.1 Forward Model

**FIGURE 1:**
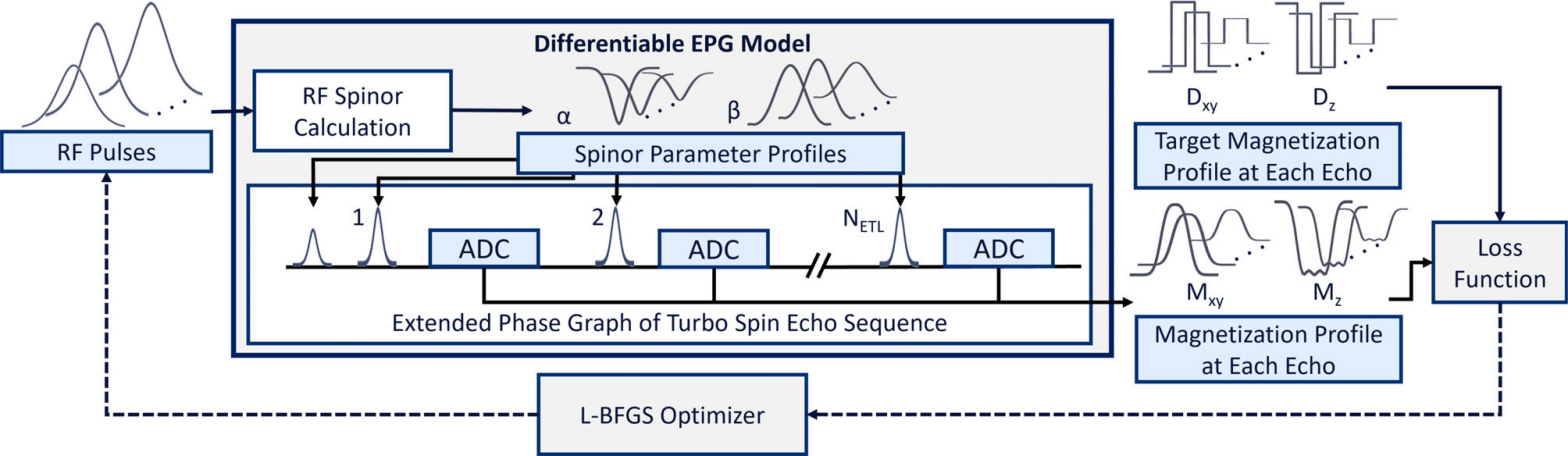
Forward model and optimization strategy to design RF pulses for slice profile consistency across echoes in TSE. The differentiable EPG model uses the spinor representations of RF pulses from the SLR algorithm to apply RF rotations. The loss function is minimized using the PyTorch L‐BFGS optimizer to design the excitation and refocusing RF pulses.

Figure 1 provides an overview of the steps in the forward model for RF pulse design. The EPG‐based forward model was implemented in PyTorch [16] using tensor operations compatible with its “autograd” functionality, which tracks all numerical operations to enable automatic differentiation and thereby provides a fully differentiable formulation without linearization. The model takes as input the excitation RF pulse, all refocusing RF pulses, the echo spacing time ($T_{\text{ESP}}$), the relaxation constants $T_{1}$ and $T_{2}$, and the spatial locations along the slice profile to calculate the resulting magnetization. The repetition time (TR) was assumed to be sufficiently long for magnetization recovery and was therefore not included in the forward model, so that it calculated signals for one echo train for one slice, unless otherwise noted. The model returned the $M_{x}$, $M_{y}$, and $M_{z}$ components of the magnetization at each echo in the sequence across the provided spatial locations, known as a partitioned extended phase graph.

The classical extended phase graph algorithm applies RF rotations using 3×3 rotation matrices [17]. In the present work, the spinor‐EPG algorithm [15] was instead used to apply rotations in SU(2) using complex‐valued spinors α and β, as in the SLR algorithm [9]. To apply 2×2 rotation matrices, the EPG states were tracked using the full ${\tilde{F}}^{+}(\pm k)$ and $\tilde{Z}(\pm k)$ representation, without explicitly tracking ${\tilde{F}}^{-}$. The hard pulse approximation was used to calculate the spinors for each RF pulse at each spatial location [9]. The resulting spinor matrices were then applied to the states as instantaneous rotations occurring in the middle of the finite RF pulse duration. The excitation RF pulse was first applied to the extended phase graph state matrix via its spinor profile representation. The following sequence of events was then repeated to obtain the magnetization at each echo time: relaxation from the previous echo time to the middle of the refocusing RF pulse, crusher gradient, refocusing RF pulse using its spinor profile representation, crusher gradient, relaxation from the middle of the refocusing RF pulse to the next echo time. The ${\tilde{F}}_{0}$ state, representing the measured complex $M_{xy}$ signal, and the ${\tilde{Z}}_{0}$ state, representing the measured $M_{z}$ signal, were recorded at each echo across all spatial locations after the second relaxation was applied.

### 2.2 RF Pulse Optimization Framework

Because the forward model was implemented using differentiable PyTorch tensor operations, the slice profile at each echo was a differentiable function of the preceding RF pulse shapes. Any differentiable loss function computed on the calculated magnetization slice profiles can then be minimized by iteratively updating the RF pulse shapes according to a nonlinear optimization algorithm. Figure 1 illustrates the structure of this optimization framework.

In addition to the sequence parameters required by the forward model, the optimization framework requires the user to specify the target $M_{xy}$ (signal) and $M_{z}$ at each echo, which can either be directly specified or calculated using a target flip angle progression for each RF pulse. In the latter case, the target echo signals can be determined by performing the EPG calculation at the center of the slice using a hard pulse with the specified flip angle train. To design pulses simultaneously for multiple $T_{1}$/$T_{2}$ pairs, the hard pulse calculation is repeated for each pair. The target echo signals are then set as the target passband values for $M_{xy}$ and $M_{z}$ at each echo.

The loss function was designed to balance slice profile similarity at each echo with achieving the target signal magnitudes. The loss was calculated on the magnetization slice profiles as:

(1) $$ℒ=\underset{Tissues}{\sum }\|M_{xy}-D_{xy}{\|}_{F,W}^{2}+\|M_{z}-D_{z}{\|}_{F,W}^{2}+\lambda \overset{N_{\text{ETL}}}{\underset{n=2}{\sum }}\sigma_{n}^{2}(M_{xy}),$$

where F denotes the Frobenius norm and $M_{xy}$ and $M_{z}$ are matrices of calculated transverse and longitudinal magnetizations whose columns span echoes and rows span spatial locations across the slice profiles. The dependency of ℒ, $M_{xy}$, $M_{z}$, and $\sigma_{n}$ (defined below) on the RF pulse shapes are suppressed for brevity. $D_{xy}$ and $D_{z}$ are matrices of target magnetization profiles for each echo. The widths of the pass‐, transition‐, and stop‐bands of the target profiles were calculated from the pulses' time‐bandwidth product, approximating the profile as the response of a linear filter [9]. The magnitudes of the target $M_{xy}$ pass‐bands were the target signal. $W_{xy}$ is an $M_{xy}$ error weighting matrix that was the same for every echo, and contained pass‐band weights of 100, transition‐band weights of 0, and stop‐band weights of 1. $W_{z}$ is an $M_{z}$ error weighting matrix that was the same for every echo, and contained pass‐band weights of 0, transition‐band weights of 0, and stop‐band weights of 1. $\sigma_{n}$ is the nth singular value of $M_{xy}$, and λ is a weighting factor. Since the EPG calculation depends on $T_{1}$ and $T_{2}$, this loss function is computed for all tissue types of interest.

The first two terms in Equation (1) measure the errors between the target and realized magnetization profiles, and are similar to those used in optimal control RF pulse design [10]. The final term is a measure of the dissimilarities of the $M_{xy}$ profiles across echoes. By penalizing the sum of squared singular values $\sigma_{n}$ for n>1, the algorithm is encouraged to find pulse shapes that produce a rank‐1 $M_{xy}$ matrix, so that all the echo profiles have the same shape but are allowed to have different scalings at each echo. Adding this term encourages the profiles to have the same ripple patterns and transition shapes, to maximally stabilize the patterns beyond simply achieving target average passband and stopband amplitudes for each echo.

The PyTorch L‐BFGS optimization algorithm [18] was applied to minimize the loss function by iteratively updating the RF pulse shapes. Each point in every pulse, including the excitation pulse and all refocusing pulses, was allowed to take on any real value during the optimization. The phases of the RF pulses were set to meet the CPMG conditions [1]. The RF pulse shapes can be initialized to a set of RF pulse shapes such as conventional sinc or SLR pulses, or to zeroes. The learning rate of the PyTorch L‐BFGS algorithm, the number of iterations, and the weighting factor λ can be manually tuned for each optimization scenario. In practice, the learning rate and number of iterations could be set by repeating the optimization across a set of potential parameter values and choosing the values that result in the smallest final loss, and λ could be set using the L‐curve method.

## 3 Methods

### 3.1 RF Pulse Designs

RF pulses were first designed for a $T_{2}$‐weighted 2D TSE sequence used clinically for standard neuroimaging on a 3T MRI scanner (Vida, Siemens Healthineers, Erlangen, Germany). The sequence used 15 echoes with an 11.1 ms echo spacing time and TR of 6 s. The flip angles were 90° for the excitation pulse, 165° for the first refocusing pulse, and 150° for all subsequent refocusing pulses, which were set to follow the rule of making the first refocusing flip angle equal to 90° plus half of the desired refocusing flip angle to stabilize echo magnitudes [3]. The time‐bandwidth product of 2 defined the main‐lobe width of the slice‐selective response, while the passband and stopband edges were determined using 1% ripple specifications as in linear‐phase FIR filter design. They were initialized to vendor‐specific shapes already used in the sequence. Each RF pulse was 128 time points long, and was later interpolated to the desired RF pulse duration for scanner implementation. The forward model and loss function were calculated for white matter ($T_{1}=832$ ms, $T_{2}=80$ ms) and gray matter ($T_{1}=1331$ ms, $T_{2}=110$ ms) [19]. The L‐BFGS optimizer was run for 15 iterations on a PC (Intel Core Ultra 9 185 H (2.30 GHz), 32 GB RAM), which took on average 25.6 s per iteration. The optimization used a learning rate of 0.1 and λ=0.2. λ was first set using an initial learning rate of 0.01, and was increased manually from 0.001 until the echo signal amplitudes began to visibly attenuate. Then, using this λ=0.2, the learning rate was chosen to minimize loss. The optimization was repeated for $N_{\text{ETL}}$ ranging from 2 to 20, with all other optimization parameters kept constant. A second design was performed to demonstrate the method's flexibility to incorporate user‐defined target signal progressions. In this design, the target signal for every echo was set to the expected signal magnitude at the ninth echo (at a TE of 100 ms) of the clinical sequence described above. The RF pulses were initialized to zero because there was no existing set of RF pulses that met this target signal progression. All other parameters were set the same as in the first design.

For comparison to a conventional pulse design method, SLR pulses were designed using PulPy [20] with the same time‐bandwidth product of 2. The excitation pulse was designed using the excitation algorithm with a linear‐phase least‐squares filter, and the refocusing pulse was designed using the spin echo algorithm with a linear‐phase least‐squares filter, both with 1% pass‐ and stopband ripple levels. The refocusing pulse was duplicated across the train, and each replicate was scaled to produce the desired flip angle in the middle of the slice.

### 3.2 Simulations

The optimized and SLR RF pulses were input into the forward EPG model with the same TSE sequence parameters to compare the resulting magnetization profiles at each echo. The z‐axes of magnetization profile plots were scaled so that the SLR and optimized pulses both had a mean full‐width at half maximum (FWHM) of 4 mm. The simulation was repeated for an interleaved multi‐slice case. The forward model was adjusted to take as input the initial $\tilde{F}$ and $\tilde{Z}$ states across space. At each slice, the simulation was performed across the same z positions. The final magnetization at the end of the standard simulation then underwent the relaxation transformation for the interslice time. Perfect spoiling of $M_{xy}$ after each echo train was assumed, which was modeled by zeroing all $\tilde{F}$ states in the EPG simulation. The remaining magnetization in $M_{z}$ was used as the initial condition for the next slice. This process was repeated for all slices in an interleaved order, with all odd slices first, followed by all even slices. The simulation was performed for 11 slices with 30% slice gap assuming white matter relaxation values until steady state was reached.

### 3.3 Phantom Slice Profile Measurement

All imaging experiments were performed on the 3T scanner using a 20‐channel phased array receive coil. Slice profiles were measured at each echo in a homogeneous spherical phantom. Pulse sequences to measure the slice profile at each echo over one TR were implemented in Pulseq [21] with $N_{\text{ETL}}=15$, $T_{\text{ESP}}=11.1$ ms, and TR =6 s. A 16 mm slice was excited, and readout was then performed at each echo along the z‐direction, with 256 readout points and a resolution of 0.5 mm. Magnitude slice profiles were reconstructed using a 1D IFFT operation followed by a sum‐of‐squares coil combination.

### 3.4 Human Brain Imaging

In vivo imaging was performed in a healthy adult male volunteer with IRB approval and volunteer consent, and in two cadaver heads with the standard and optimized RF pulses corresponding to a 150° flip angle for the first refocusing pulse and 165° flip angle for the remaining refocusing pulses. All sequences were implemented in Pulseq. Single‐slice brain 2D TSE images were acquired with $N_{\text{ETL}}=15$, $T_{\text{ESP}}=11.1$ ms, TR =6 s, ${\text{TE}}_{\text{eff}}=100$ ms, FOV =22×22 ${\text{cm}}^{2}$, matrix size =512, and slice thickness =4 mm. No acceleration was applied, and the total imaging time was 3 min and 30 s. The phase encode direction was from posterior to anterior. An additional interleaved multi‐slice acquisition was performed with the same sequence parameters and 11 axial slices. The slice gap was set to 30% of the slice thickness such that the distance between the centers of adjacent slices was 5.2 mm. Images were reconstructed using an IFFT operation followed by Walsh coil combination [22].

### 3.5 $T_{2}$ Mapping

A MESE sequence corresponding to the TSE sequence was implemented in Pulseq for $T_{2}$ mapping with $N_{\text{ETL}}=15$, $T_{\text{ESP}}=11.1$ ms, TR =6 s, FOV =22×22 ${\text{cm}}^{2}$, matrix size =128, and slice thickness =4 mm. The total imaging time was 12 min and 54 s. Measurements were collected in a NIST phantom [23] using the $T_{2}$ ${\text{MnCl}}_{2}$ array and in vivo in a healthy adult male volunteer with IRB approval and volunteer consent. $T_{2}$ maps were calculated using open‐source software [14], wherein the $T_{2}$ of each voxel was determined by performing an optimization in PyTorch that iteratively updated $T_{2}$ to minimize the difference between the measured and predicted signal magnitude at each echo. The predicted signal magnitude was calculated with a standard, differentiable EPG model (not slice‐selective) given the flip angles achieved at the center of the slice by the pulses.

A single spin echo sequence with a slice‐selective SLR excitation pulse and nonselective $180^{\circ }$ hard refocusing pulse was used to calculate a reference $T_{2}$ value for each ROI in the NIST phantom with TEs = [15, 30, 45, 60, 90, 150, 200] ms, TR = 6 s, and total acquisition time = 90 min and 18 s. To calculate a reference $T_{2}$ map in vivo, a MESE sequence was implemented in Pulseq with the same parameters used for the SLR and optimized MESE sequences, except with a slice‐selective SLR excitation pulse and nonselective $180^{\circ }$ hard pulses for all refocusing pulses. For both reference methods, the measured signal at each pixel was fit to an exponential decay curve in Matlab.

## 4 Results

### 4.1 Simulations

**FIGURE 2:**
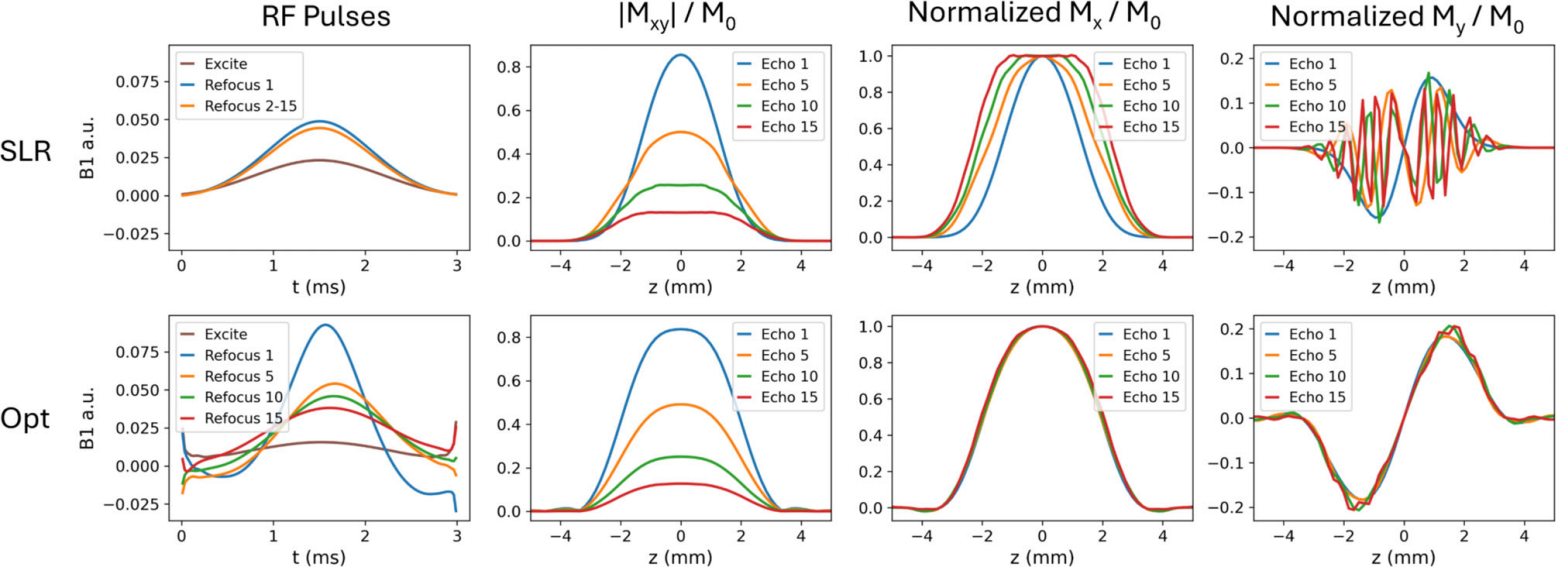
Optimized RF pulses (bottom) compared to SLR pulses (top) in simulation. The $\left|M_{xy}\right|$ and normalized $M_{x}$ and $M_{y}$ magnetization profiles subsequent to the refocusing pulses for echoes 1, 5, 10, and 15 are plotted, for white matter relaxation values ($T_{1}$ = 832 ms, $T_{2}$ = 80 ms).

Figure 2 shows SLR and optimized RF pulse shapes for $N_{\text{ETL}}=15$ echoes, along with their magnetization profiles in the slice direction across echoes. To produce matching slice widths between SLR and optimized pulses, the slice select gradient amplitude was adjusted assuming a time bandwidth product of 1.825 for the SLR pulses, and 1.5 for the optimized pulses in both these simulations and in imaging experiments. Given these time bandwidth products and assuming a slice width of 4 mm, the full widths at half maximum of the simulated slice profiles ranged from 2.81 mm to 4.66 mm for the SLR pulses, and 4.00 mm to 4.17 mm for the optimized pulses. Though the SLR refocusing pulses were scalar versions of each other, the slice profiles they produced widened across echoes, likely due to stimulated echoes. Their $M_{y}$ profiles varied between echoes. In contrast, while the optimized RF pulses had distinct shapes, their slice widths remain approximately constant across echoes, and their $M_{y}$ profiles are approximately constant as well.

The optimized pulses generally have higher amplitudes at higher spatial frequencies at their beginning and end, to compensate for the increasingly high frequency energy content in the magnetization slice profiles preceding each pulse. The peaks at the edges of the pulses are not numerical artifacts introduced in the optimization, but rather are key to achieving this high similarity. As shown in Supporting Information Figure S1, truncating the RF pulses to remove peaks at the ends results in oscillations in the magnetization profiles and more variable slice widths. The design loss (Equation 1) for the truncated pulses was 3.1 times larger than the loss for the original optimized pulses.

Though the target echo signals used to design the optimized pulses were calculated using hard pulses with the same flip angle progression as the SLR pulses, Supporting Information Figure S2 shows that the integrated $B_{1}$ of the optimized pulses, which determines the flip angle in the center of the slice, deviated from the SLR pulses in order to achieve the desired target signal. In particular, the integrated $B_{1}$ of the optimized refocusing RF pulses increased for the last few echoes. The optimized pulses also have a different $B_{1}$ RMS compared to the SLR pulses, as shown in Figure S2, where the first pulses in particular had a higher peak $B_{1}$. The SAR of the optimized pulses was in total 32.1% higher than the SLR pulses. This increase could be mitigated by lengthening the pulses by 15.0%, though this may increase $T_{2}$ blurring if the echo spacing time is short and needs to be lengthened to accommodate the longer pulse. Alternatively, the time‐bandwidth product of the SLR pulses could be increased to 2.3 to match the SAR of the optimized pulses. The slice profile simulation results using such SAR‐matched SLR pulses are described below.

**FIGURE 3:**
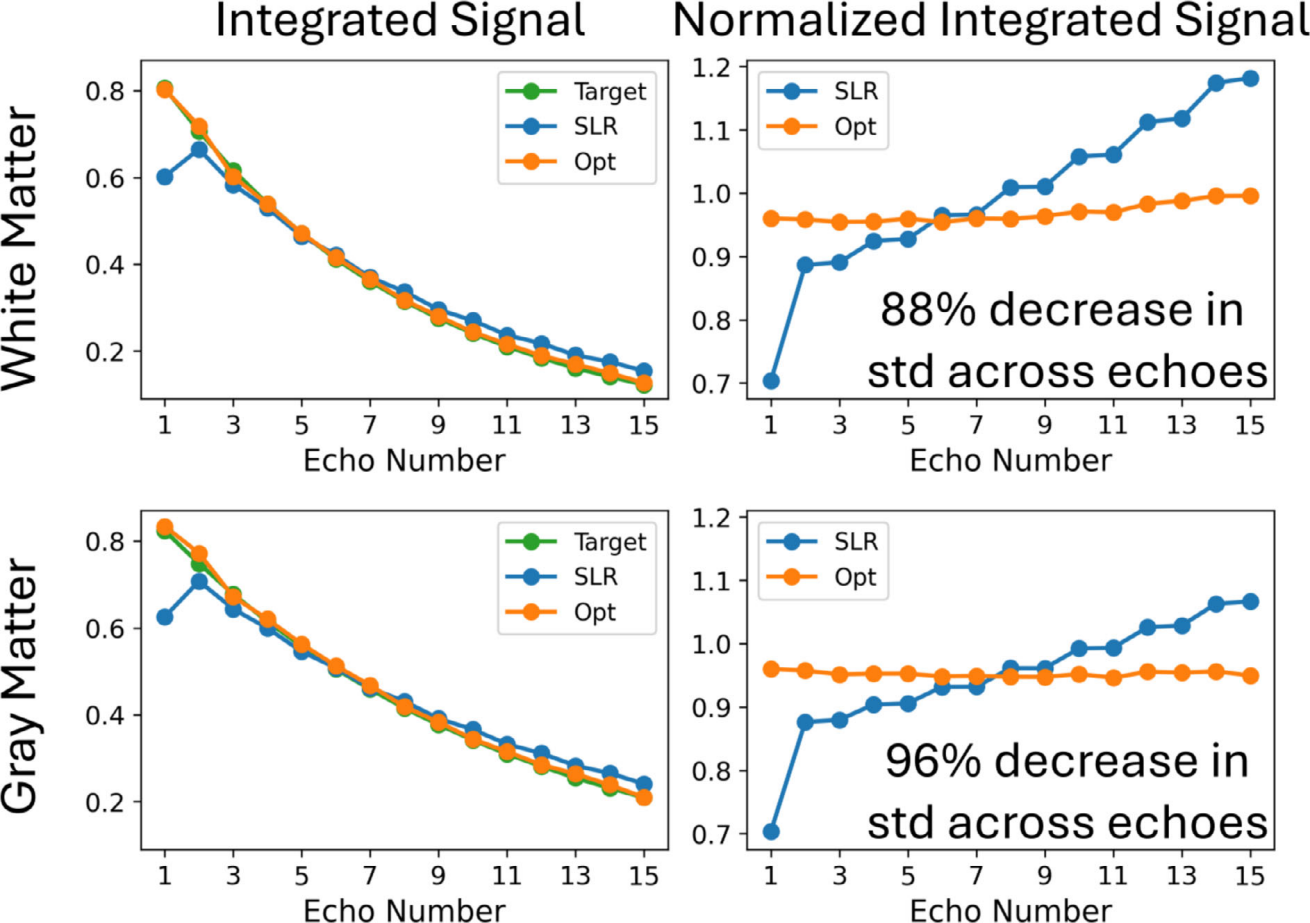
Left: Integrated transverse magnetization (signal) across the slice at each echo, for white matter (top) and gray matter (bottom). The target signal used in the optimization is also plotted, which was calculated using hard pulses with the same flip angle progression as the SLR pulses. Right: Normalized integrated signals at each echo, calculated by dividing the integrated signal by the echo amplitude at the center of the slice. Decreases in the standard deviations of the normalized integrated complex magnetization profiles with optimized pulses are reported.

**FIGURE 4:**
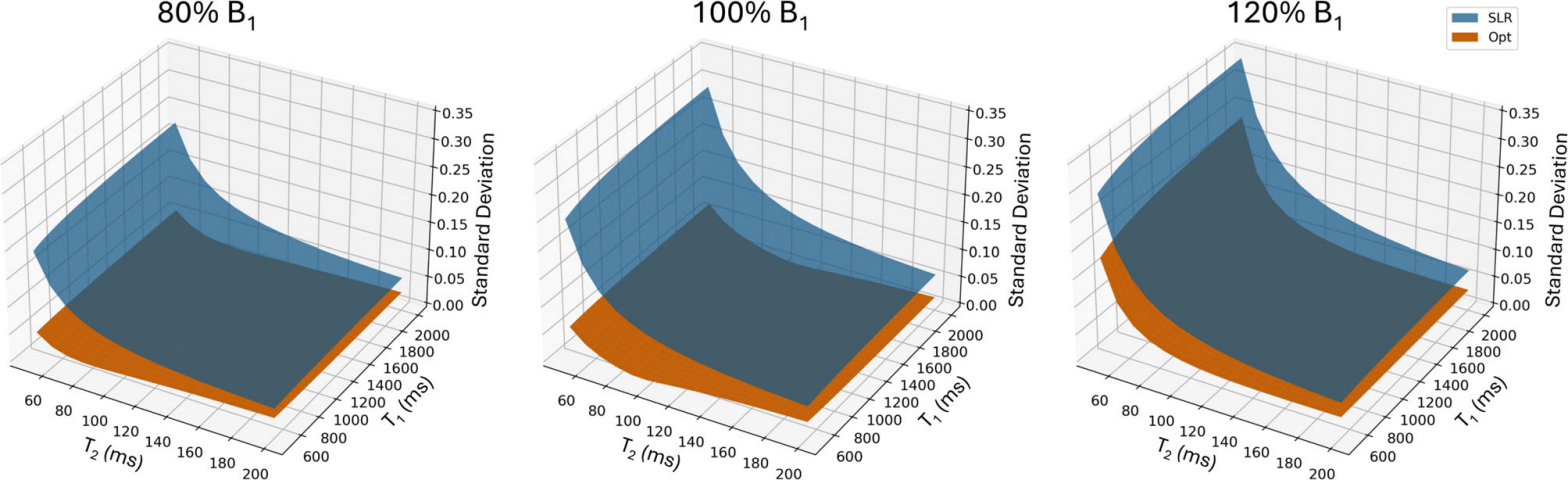
Standard deviations across echoes of the integrated normalized signals versus $T_{1}$ (500–2000 ms) and $T_{2}$ (50–200 ms) for 80% (left), 100% (center), and 120% (right) of nominal $B_{1}$. For all three $B_{1}$ values, the standard deviation is always lower for the optimized pulses (orange) versus SLR pulses (blue).

Figure 3 plots the integrated and normalized integrated signal profiles for white and gray matter at each echo for SLR and optimized pulses, where the normalized integrated signals were calculated by dividing the integrated signals by the signal amplitude at the center of the slice at each echo. The RMSE between the simulated integrated signal and the target signal decreased by 89% for white matter (0.0585 vs 0.0067) and 84% for gray matter (0.0565 vs 0.0088) using the optimized pulses. Even though the SLR pulses use the same flip angle train as the target signal, slice widening across echoes caused the integrated signal to vary from the target. This variability is further reflected in the normalized integrated signals, where the standard deviations of the normalized optimized integrated signals across echoes were 88% lower than SLR for white matter (0.1262 vs 0.0149) and 96% lower for gray matter (0.0918 vs 0.0041). Supporting Information Figure S3 shows that the SAR‐matched SLR pulses with time‐bandwidth equal to 2.3 result in somewhat sharper profiles as expected, which also improved the RMSE between the simulated integrated signal and the target signal, and the slice profile similarity compared to the time‐bandwidth 2 SLR pulses. The RMSE between the simulated integrated signal and the target signal for the SAR‐matched SLR pulses was 0.0455 for white matter and 0.0437 for gray matter, which still correspond to an 85% and 80% lower RMSE using the optimized pulses, respectively. The standard deviation between echoes of the normalized integrated magnetization for the SAR‐matched SLR pulses was 0.0972 for white matter and 0.0716 for gray matter, which means the optimized RF pulses still resulted in 85% lower standard deviation for white matter and 94% for gray matter. Figure 4 shows normalized integrated signal standard deviations for optimized and SLR pulses across $T_{1}$, $T_{2}$, and $B_{1}$ dimensions. Though the optimization only used $T_{1}$'s and $T_{2}$'s of white and gray matter while assuming a single nominal $B_{1}$ amplitude, these results show that their performance generalizes away from these assumed values, and that their standard deviations were always lower than SLR. Specifically the average percent decrease in the standard deviation for the optimized pulses is 66.5±12.0% at 80% nominal $B_{1}$, 82.3±9.1% at 100% nominal $B_{1}$, and 53.8±6.5% at 120% nominal $B_{1}$.

**FIGURE 5:**
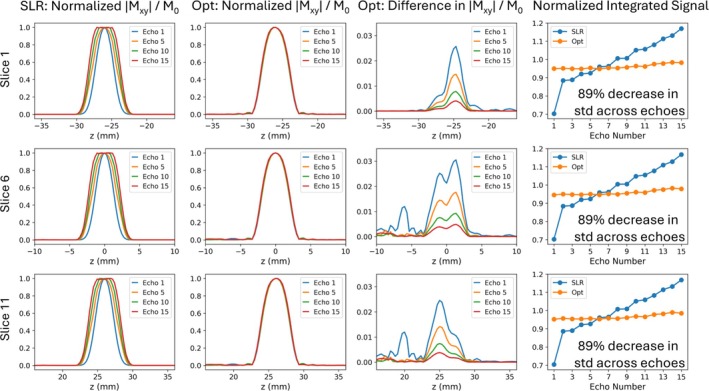
Simulated signal profiles normalized by echo amplitude across echoes for three slice positions of an eleven‐slice interleaved acquisition. The multi‐ slice $M_{xy}/M_{0}$ difference profiles for the optimized RF pulses were calculated by taking the difference between the slice profiles in the single‐slice and multi‐slice cases. Normalized integrated signals at each echo are plotted on the right, which were calculated by dividing the integrated signal by the echo amplitude at the center of the slice. Decreases in the standard deviations of the normalized integrated complex magnetization profiles with optimized pulses are reported on those plots.

Figure 5 compares the performance of the SLR and optimized pulses in an interleaved multi‐slice sequence with 11 slices. In the optimized case, perturbation of magnetization between slices increased signal ripples outside the slice up to 1% of the target signal in early echoes. These ripples were attenuated in later echoes, and the overall RMSE's between the single‐slice and multi‐slice profiles for the displayed slices were 0.0001 for Slice 1, 0.0116 for Slice 6, and 0.0004 for Slice 11. Despite these differences between single‐slice and multi‐slice signal profiles, the normalized integrated signal plots in Figure 5 show that the optimized pulses maintained consistent signal slice profiles between echoes.

Supporting Information Figure S4 illustrates how RF pulses designed for different $N_{\text{ETL}}$ vary. Figure S4a plots the ratio of design loss (Equation 1) for each $N_{\text{ETL}}$ when it is evaluated with pulses designed for $N_{\text{ETL}}=20$, divided by the loss for pulses designed specifically for that $N_{\text{ETL}}$. The ratios are close to one for trains with more than eight echoes, indicating that the pulse shapes vary less between $N_{\text{ETL}}$'s, while for smaller $N_{\text{ETL}}$'s the ratio is up to ten times larger, indicating that it is best to use pulses designed directly for these echo trains. Figure S4b shows the RF pulse shapes and magnetization profiles for $N_{\text{ETL}}=10$ and 20, for echoes 1, 5, and 10. The RF pulses optimized specifically for $N_{\text{ETL}}=10$ have a wider passband and smaller $M_{y}$ than RF pulses designed for $N_{\text{ETL}}=20$, without higher peak amplitudes. This superior result reflects the algorithm's ability to generate a more optimal result with fewer echoes because there are fewer constraints in the design problem.

Supporting Information Figure S5a shows the pulses and magnetization profiles for the second set of pulses designed to produce a constant signal amplitude across echoes. The RF pulses have lower integrated $B_{1}$ than the first pulses designed for varying signal amplitudes, since the target signal was lower for earlier echoes. Even with this change in the target signal progression, the magnetization profiles are consistent between echoes, as reflected in the close agreement in the normalized $M_{x}$ and $M_{y}$ components. Figure S5b further shows that the pulses' performance is consistent for white and gray matter.

### 4.2 Phantom Slice Profile Measurement

**FIGURE 6:**
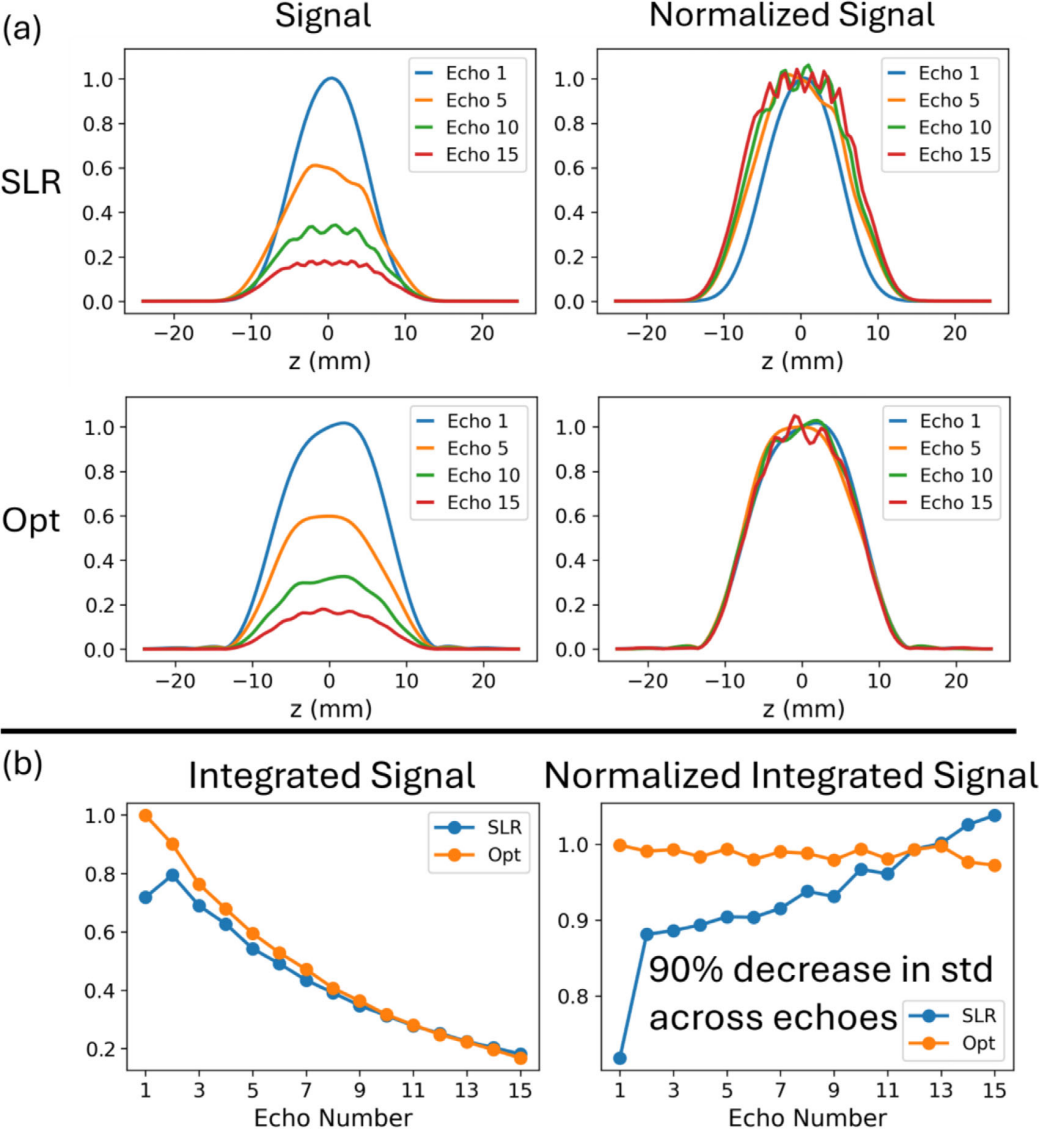
(a) Measured slice profiles from four echoes of a TSE sequence in a homogeneous phantom using SLR RF pulses (top) and optimized RF pulses (bottom), without (left) and with (right) normalization by the center profile amplitude. (b) Integrated (left) and normalized integrated (right) signal across the slice at each echo is plotted for the optimized and SLR pulses (left). The reduction in standard deviation of the normalized integrated signals across echoes for optimized pulses is reported.

Figure 6a shows slice profiles measured in a homogeneous phantom using SLR (top) and optimized (bottom) pulses. Visually, the shapes of the measured slice profiles from the optimized pulses are more consistent between echoes. The slice does not widen across echoes as it does for the SLR pulses, and there are smaller ripples across the passband of the slice profiles. The FWHMs of the slice profiles ranged from 11.125 mm to 16.000 mm for the SLR pulses, compared to 15.250 mm to 15.875 mm for the optimized pulses. Figure 6b plots integrated and normalized integrated signals across the slice between echoes, which agree with the simulation results in Figure 3. The standard deviation of the normalized integrated signals across echoes was 90% lower with the optimized pulses (0.0777 vs 0.0081). Supporting Information Figure S6 shows the measured slice profiles and integrated signals from the RF pulses optimized for constant signal amplitude. The signal amplitude varies little between echoes, and the profile shapes are consistent.

### 4.3 Human Brain Imaging

**FIGURE 7:**
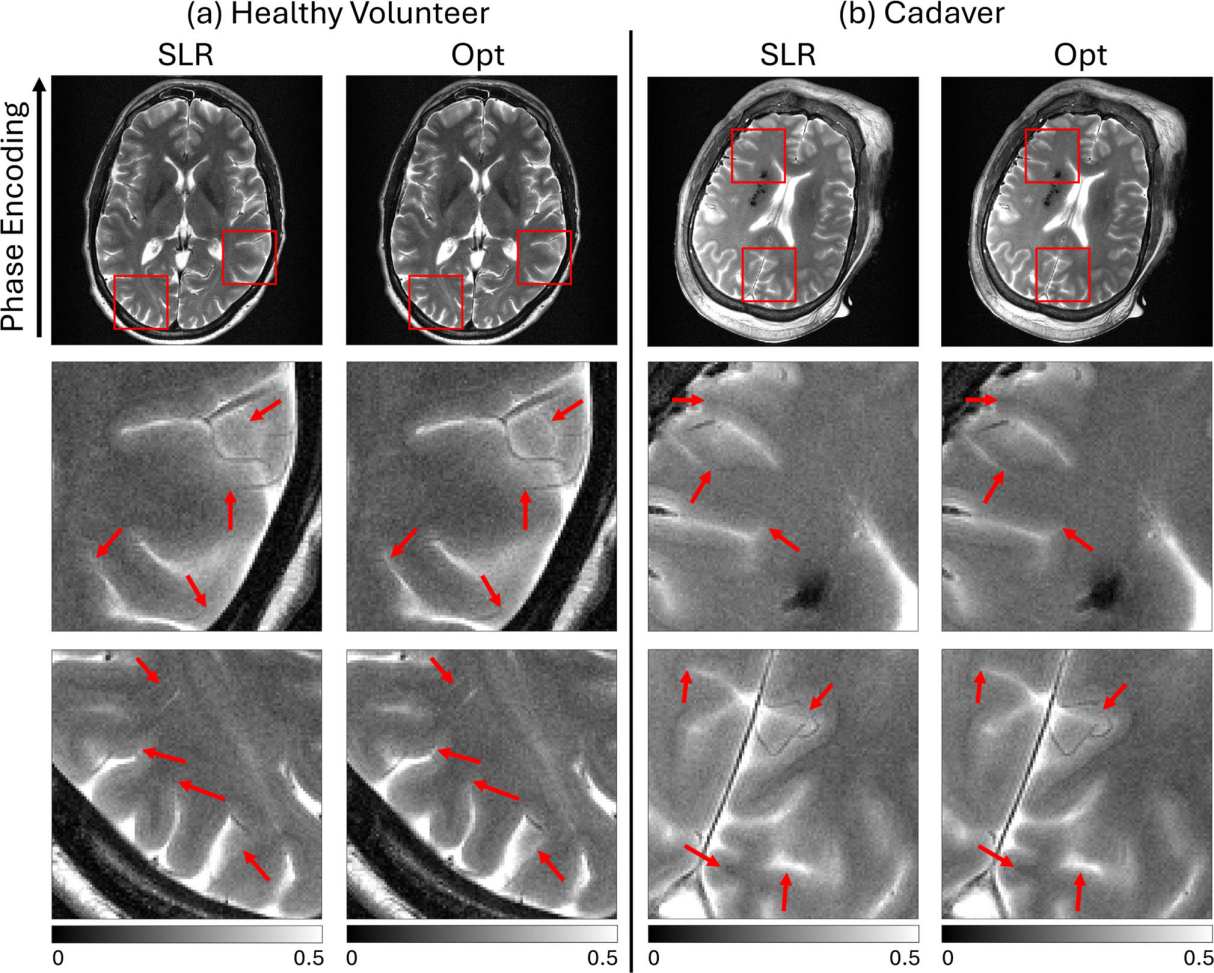
(a) $T_{2}$‐weighted single‐slice TSE head images from a healthy volunteer using SLR (left) and optimized (right) RF pulses. Red boxes in the top images show the locations of the regions of interest below. Red arrows point to areas of increased sharpness and conspicuity using the optimized pulses, primarily in the phase‐encoded direction affected by variations in slice profile. (b) Cadaver brain images using the SLR (left) and optimized (right) RF pulses. Red arrows again highlight areas of increased sharpness using the optimized pulses.

**FIGURE 8:**
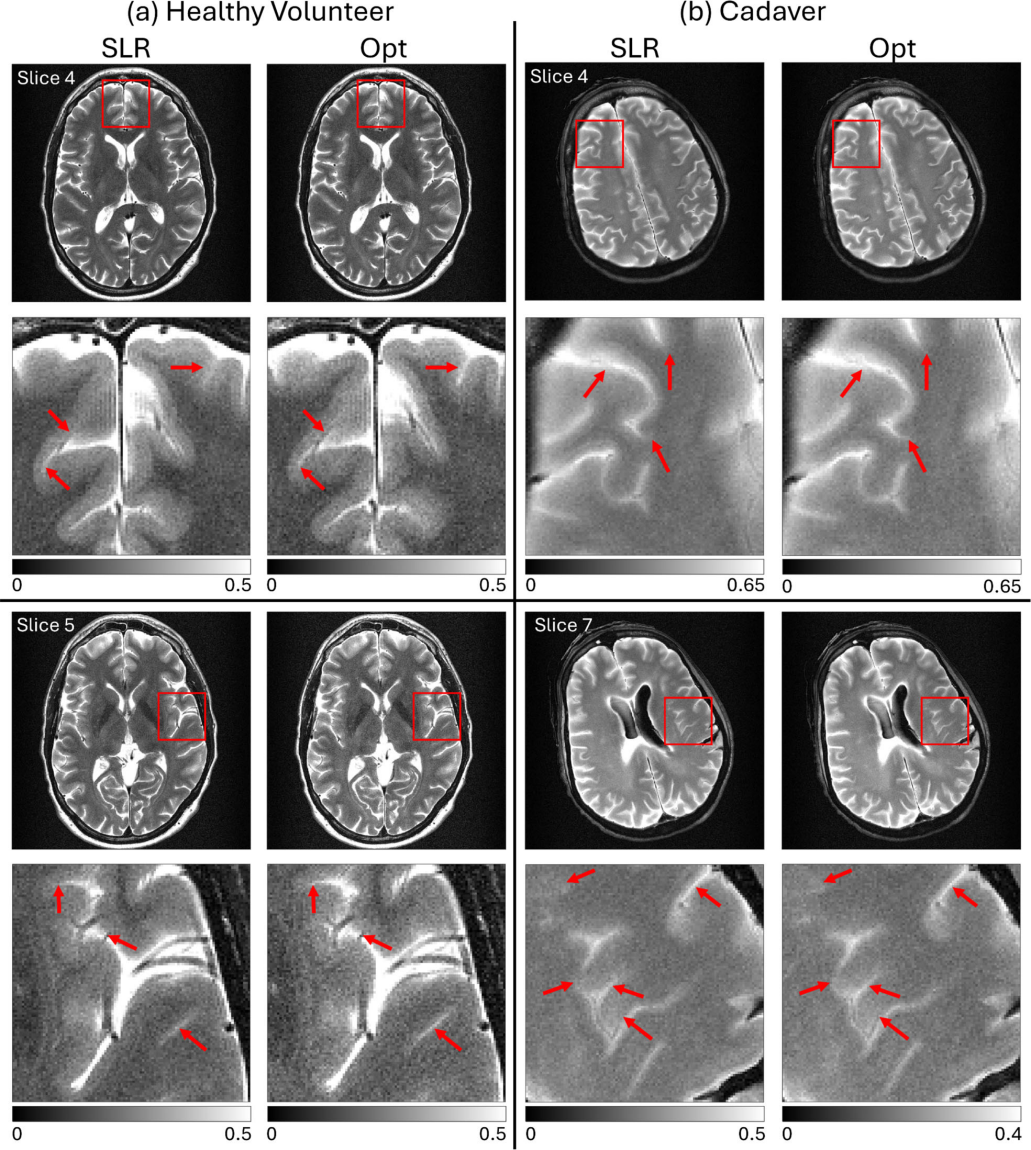
(a) $T_{2}$‐weighted multi‐slice TSE head images from a healthy volunteer using SLR (left) and optimized (right) RF pulses. The sequence had 11 total slices, and slice 4 (top) and slice 5 (bottom) are shown. Red boxes show the locations of the regions of interest below. Red arrows point to areas of increased sharpness in the optimized images. (b) Cadaver brain images using the same eleven‐slice TSE sequence with SLR (left) and optimized (right) RF pulses. Slice 4 (top) and slice 7 (bottom) are shown.

Figure 7 shows $T_{2}$‐weighted TSE images acquired using the SLR and optimized pulses in a volunteer (a) and a cadaver (b). The same slice was selected for SLR and optimized scans. Areas of increased sharpness are indicated by red arrows in the optimized images, particularly around the CSF and veins perpendicular to the phase encode direction, which is the direction prone to blurring due to variations in signal across echoes. The cadaver images demonstrate that these improvements hold in the absence of the possibility of motion between the scans. Figure 8 further shows $T_{2}$‐weighted multi‐slice TSE images acquired using the SLR and optimized pulses in a volunteer (a) and a cadaver (b), which shows that the improvements with optimized pulses extend to the multi‐slice imaging case as well.

### 4.4 $T_{2}$ Mapping

**FIGURE 9:**
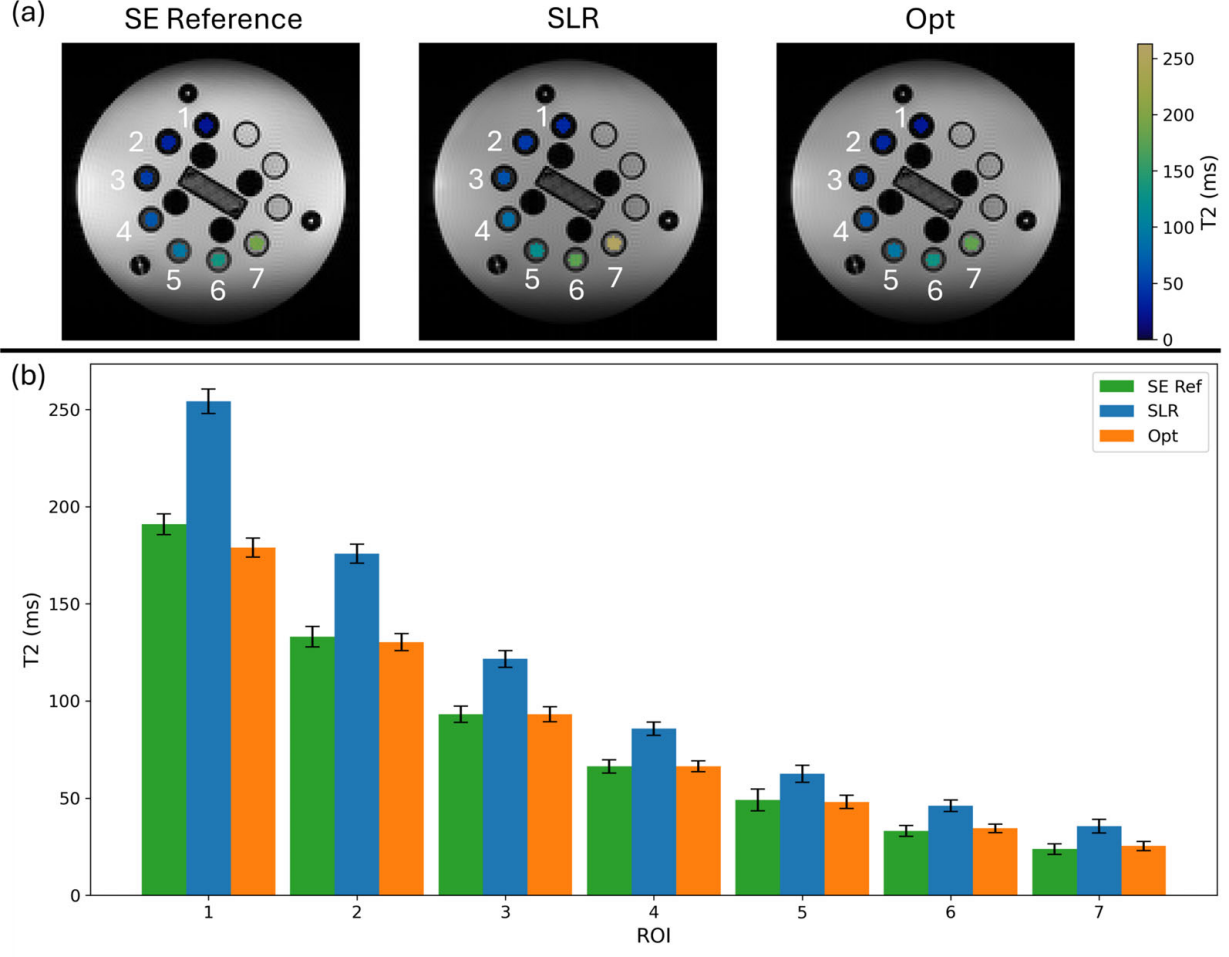
(a) Calculated $T_{2}$ values in the NIST phantom using the reference method, SLR RF pulses, and optimized RF pulses for each pixel in seven ROIs corresponding to a $T_{2}$ range relevant for neuroimaging. The background image was reconstructed using data collected at the 8th echo during the MESE sequence for the SLR and optimized RF pulses. (b) Mean calculated $T_{2}$ value for each ROI in the NIST phantom array from the hard pulse reference method compared to mean calculated values for SLR and optimized RF pulses. Error bars correspond to one standard deviation. The seven ROIs correspond to the labels in (a).

Figure 9a shows $T_{2}$ maps in 7 ROIs of the NIST phantom's $T_{2}$ array plane, calculated from the hard pulse reference method (left) and the MESE data acquired using the SLR (center) and optimized (right) RF pulses. These ROIs were chosen because the $T_{2}$ values were between 0 and 250 ms, relevant to neuroimaging. The background images were reconstructed from the data corresponding to the eighth echo for all phase encode lines (not a TSE acquisition). The $T_{2}$ maps show that the SLR pulses lead to a consistently higher $T_{2}$ estimate than the optimized pulses. This behavior is expected, as the narrow slice profiles of early echoes bias the $T_{2}$ estimates towards larger values. Figure 9b confirms this by showing the mean estimated $T_{2}$ value for each ROI, compared to the true value calculated from the spin echo reference data. This figure shows that the SLR pulses consistently overestimate the reference $T_{2}$ value. At $T_{2}$ values closer to the values used in the optimization, the optimized pulses provide the most accurate estimates. The average percent error in $T_{2}$ across the seven ROIs was 3.0% for the optimized pulses, compared to 34.5% for the SLR pulses.

**FIGURE 10:**
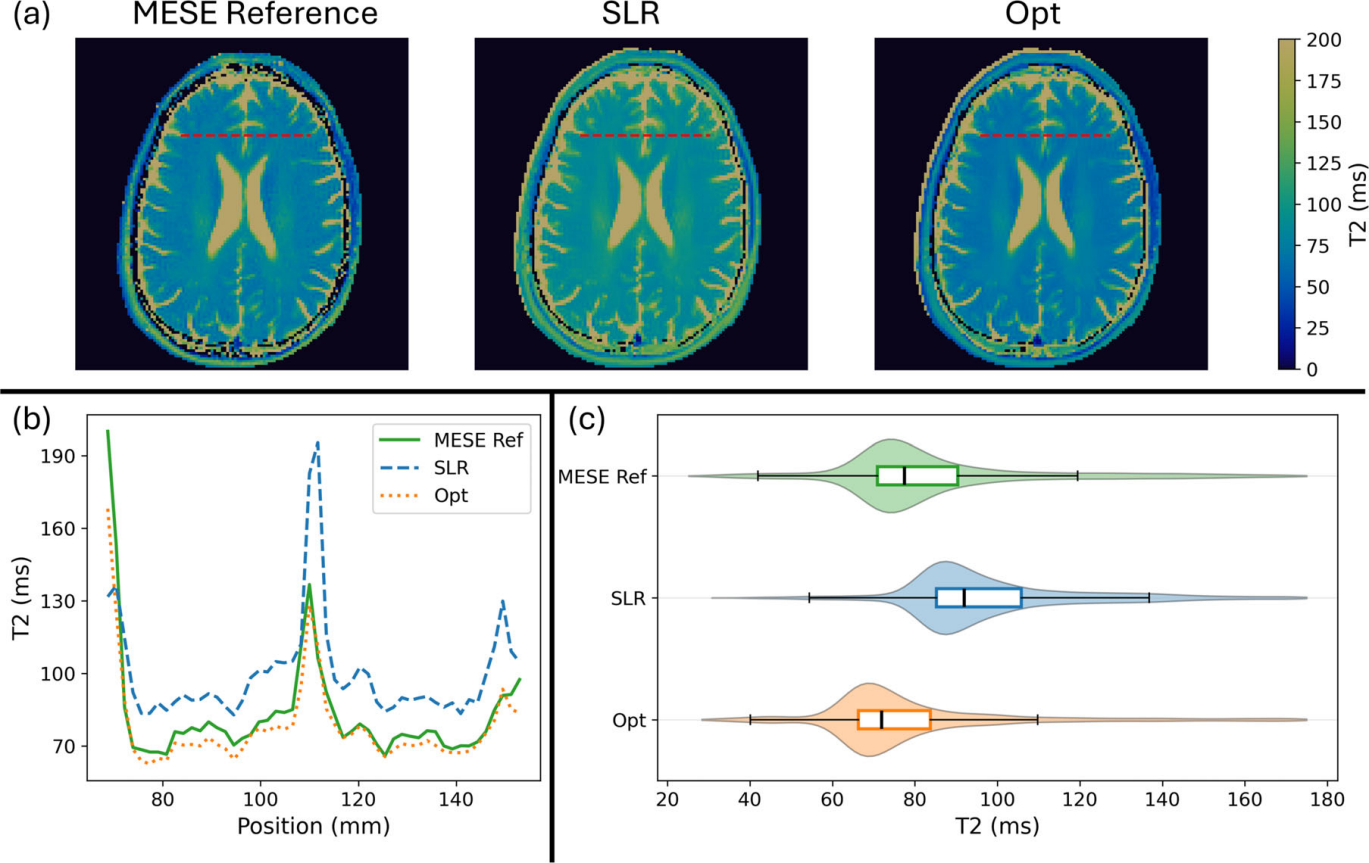
(a) Calculated $T_{2}$ maps in the head of a healthy volunteer using the MESE reference sequence, SLR pulses, and optimized pulses. (b) Calculated $T_{2}$ values along the dashed red line in (a) for MESE reference, SLR pulses, and optimized pulses. (c) Distribution of $T_{2}$ values in the brain between 25 ms and 175 ms calculated using the MESE reference, SLR pulses and optimized pulses.

Figure 10a shows $T_{2}$ maps in a transverse slice of the brain acquired in a volunteer with the hard pulse MESE reference sequence, SLR MESE sequence, and optimized MESE sequence. The $T_{2}$ maps show that the SLR pulses overestimate $T_{2}$ in vivo, as expected, wheras the optimized pulses provide a more accurate estimate compared to the MESE reference. Figure 10b shows the 1D $T_{2}$ profile along the dashed red line in the images. The RMSE between the reference profile and optimized profile decreased by 68% compared to the SLR profile (from 24.8 to 8.0). Figure 10c shows the distribution of $T_{2}$ values in the brain between 25 and 175 ms, chosen to exclude CSF. The median and 1st and 3rd quartiles for the MESE reference were 77.5 ms, 71.0 ms, and 90.4 ms, respectively. The optimized pulses resulted in median and 1st and 3rd quartiles of 71.9 ms, 66.3 ms, and 83.7 ms, respectively, and the SLR pulses resulted in 92.0 ms, 85.2 ms, and 106.2 ms, respectively. The optimized pulses decreased the error in median $T_{2}$ by 62%.

## 5 Discussion

We reported an RF pulse design method that enables optimization for consistent slice profiles in multi‐RF pulse sequences, by accounting for the evolving magnetization pathways throughout the sequence. Unlike conventional pulse design methods, the method does not require the assumption that the magnetization is in the same state prior to each RF pulse or that the slice profile shape will scale linearly with the flip angle. Results from simulations and phantom experiments showed that this strategy achieved both the target signal TSE progression and more consistent slice profiles across echoes, compared to RF pulses designed using the SLR algorithm. Single‐slice and multi‐slice human brain images in a volunteer and cadaver showed that the optimized RF pulses reduced blurring in the phase encode direction, without modifying contrast compared to SLR pulses. Since the introduction of optimized pulses did not significantly affect the overall rate of signal decay across echoes (Figure 3), it is likely that the reduced blurring with optimized pulses is due to the consistent slice profiles they generate, in contrast to the SLR pulses which produced widening slice profiles across the train that increasingly averaged adjacent features into the slice. Optimized RF pulses also reduced $T_{2}$ estimate error in phantom and human brain $T_{2}$ mapping using an EPG amplitude fit compared to SLR pulses, which overestimate $T_{2}$ due to the slice widening between echoes. SLR pulses would instead require a refocusing width that is approximately three times wider than the excite width to achieve accurate $T_{2}$ estimates, limiting the use of multi‐slice MESE $T_{2}$ mapping [24]. In vivo $T_{2}$ mapping used a MESE reference to limit scan time and reduce motion instead of a spin echo reference which may have introduced errors from stimulated echoes in the reference $T_{2}$ values.

Slice profile consistency held across a wide range of expected $T_{1}$, $T_{2}$, and relative $B_{1}$ values. This flexibility suggests that RF pulses optimized using this method do not need to be redesigned between patients or for different sequence timing, provided the TR is sufficiently long for recovery. Pulses designed for a specific $N_{\text{ETL}}$ outperformed using a subset of pulses designed for a larger $N_{\text{ETL}}$, however the tradeoffs incurred in the optimization such as narrower profiles and increased $M_{y}$ amplitude may be acceptable, meaning it may not be necessary to redesign pulses for different $N_{\text{ETL}}$ values. Adjusting the target signal progression to change the image contrast does require a new optimization, since this would lead to a change in the target flip angle for each refocusing pulse. The computation involved in the RF pulse design may not be prohibitive to perform online; the designs performed in this work took approximately six minutes on a single CPU, but RF pulse simulations are highly amenable to parallelization across the spatial partitions, so improvements in optimization time could be achieved by executing on a GPU.

Although blurring improvements were seen in multi‐slice imaging with the optimized pulses, the forward model used in the optimization assumed a single‐slice acquisition. Further imaging improvements may be seen by performing the optimization directly on the multi‐slice forward model, at the expense of increased computation. Future work could also incorporate magnetization transfer effects into the multi‐slice forward model, such as in EPG‐X [25], which are known to change signal contrast in multi‐slice TSE images [26].

It is possible that this optimization could be applied to design pulses with the same effective blurring level as SLR pulses but with a lower time bandwidth product to instead reduce SAR. The RF pulse design approach proposed here could be combined with variable flip angle optimization schemes for 2D TSE and HASTE [27], which balance SNR, contrast, and $T_{2}$ blurring [2, 5, 8]. The optimized flip angle progression could then be used to optimize RF pulses that excite consistent slice profiles and achieve the target signal progression, which may result in further improvements in image quality by eliminating slice profile effects. This optimization could also be adapted to other echo train sequences that experience artifacts from slice profile effects by switching the order and timing of the forward model components. For instance, slice profile effects cause significant ghosting artifacts in multi‐shot spin echo EPI [28] and introduce errors in $T_{2}$ estimates in magnetic resonance fingerprinting when not properly included in dictionary generation [15, 29, 30]. This optimization approach could specifically design RF pulses with consistent echo shapes to greatly reduce slice profile effects in both sequences.

## 6 Conclusion

We have developed a method to jointly design selective RF pulses in TSE sequences for a given target signal progression with consistent slice profiles based on a differentiable extended phase graph model. Compared to SLR pulses, the optimized RF pulses increased slice profile consistency across echoes in simulation and phantom measurements, improved image sharpness in vivo, and reduced $T_{2}$ mapping error in the NIST phantom and in vivo.

## Funding

This work was supported by NIH grants R01 CA 281043, R01 EB 019437, T32 EB 007509; NSF GRFP 2437833; and Siemens Healthineers.

## Conflicts of Interest

Madison Augelli, Mark Griswold, William Grissom, and Anuj Sharma are part of the Case MRI research group, which receives support from Siemens Healthineers.

## Supporting information




**Figure S1**. Optimized RF pulses were truncated by removing four time points on both sides to eliminate peaks. The excitation pulse and four refocusing pulses are shown. Normalized $M_{x}$ and $M_{y}$ components at the four corresponding echoes are also shown for white matter relaxation values ($T_{1}$ = 832 ms, $T_{2}$ = 80 ms) to visually assess similarity in shape between echoes.
**Figure S2**. Comparison of the excitation (echo number = 0) and refocusing RF pulse integrated $B_{1}$ (left) and $B_{1}$ RMS (right) for the SLR and optimized pulses.
**Figure S3**. (a) SLR RF pulses with time‐bandwidth product 2.3 to match SAR of optimized RF pulses. Simulated magnetization profiles are plotted for white matter relaxation values ($T_{1}$ = 832 ms, $T_{2}$ = 80 ms). The excitation pulse, four representative refocusing pulses, and subsequent echo magnetization profiles from the echo train are shown, without and with normalization to their peak amplitude. (b) Slice‐integrated signals across echoes for white and gray matter, without (left) and with (right) normalization to their peak amplitude.
**Figure S4**. Dependence of RF pulse solutions on echo train length ($N_{\text{ETL}}$). (a) Ratios of design loss (Equation 1) versus $N_{\text{ETL}}$ when pulses designed for $N_{\text{ETL}}=20$ are used for shorter train lengths. Each ratio was calculated as the loss incurred using the twenty‐echo pulses at each shorter $N_{\text{ETL}}$, divided by the loss for pulses directly optimized for each $N_{\text{ETL}}$. (b) Comparison of the excitation and four refocusing RF pulse shapes designed for $N_{\text{ETL}}=10$ (top) versus $N_{\text{ETL}}=20$ (bottom), and their subsequent magnetization profiles in white matter.
**Figure S5**. (a) Optimized RF pulses designed to maintain a constant signal equal to the target signal at the ninth echo, corresponding to an effective TE of 100 ms. Simulated magnetization profiles are plotted for white matter relaxation values ($T_{1}=832\;\text{ms},T_{2}=80\;\text{ms}$). The excitation pulse, four representative refocusing pulses, and subsequent echo magnetization profiles from the echo train are shown, without and with normalization to their peak amplitude. (b) Slice‐integrated signals across echoes for white and gray matter, without (left) and with (right) normalization to their peak amplitude. Decreases in the standard deviations of the normalized integrated complex magnetization profiles with optimized pulses are reported.
**Figure S6**. (a) Measured slice profiles from a TSE sequence in a homogeneous phantom using RF pulses optimized to maintain a constant signal equal to that expected at the ninth echo. Four representative echoes are shown (left). To visually compare similarity, each echo was also scaled based on its center value (right). (b) The integrated signal across the slice at each echo (left) and normalized integrated signal (right), calculated by dividing by the echo amplitude, is plotted for the optimized pulses.

## Data Availability

The optimization, TSE pulse sequence generation, and TSE reconstruction code are hosted at https://github.com/grissomlab/tseOptRF. The experimental data, images, and additional code from this study are available upon request to the corresponding author.
