# Supplementary material for: Optimizing Selective RF Pulses for Enhanced Signal Stability in Turbo Spin Echo Using a Differentiable Extended Phase Graph Model
Source: Magn Reson Med. 2026 Mar 12;96(1):214–26. doi: 10.1002/mrm.70340 (PMC13156457; doi:10.1002/mrm.70340)
Supplement: Supplementary file 1 — Figure S1. Optimized RF pulses were truncated by removing four time points on both sides to eliminate peaks. The excitation pulse and four refocusing pulses are shown. Normalized Mx and My components at the four corresponding echoes are also shown for white matter relaxation values (T1 = 832 ms, T2 = 80 ms) to visually assess similarity in shape between echoes. Figure S2. Comparison of the excitation (echo number = 0) and refocusing RF pulse integrated B1 (left) and B1 RMS (right) for the SLR and optimized pulses. Figure S3. (a) SLR RF pulses with time‐bandwidth product 2.3 to match SAR of optimized RF pulses. Simulated magnetization profiles are plotted for white matter relaxation values (T1 = 832 ms, T2 = 80 ms). The excitation pulse, four representative refocusing pulses, and subsequent echo magnetization profiles from the echo train are shown, without and with normalization to their peak amplitude. (b) Slice‐integrated signals across echoes for white and gray matter, without (left) and with (right) normalization to their peak amplitude. Figure S4. Dependence of RF pulse solutions on echo train length (NETL). (a) Ratios of design loss (Equation 1) versus NETL when pulses designed for NETL=20 are used for shorter train lengths. Each ratio was calculated as the loss incurred using the twenty‐echo pulses at each shorter NETL, divided by the loss for pulses directly optimized for each NETL. (b) Comparison of the excitation and four refocusing RF pulse shapes designed for NETL=10 (top) versus NETL=20 (bottom), and their subsequent magnetization profiles in white matter. Figure S5. (a) Optimized RF pulses designed to maintain a constant signal equal to the target signal at the ninth echo, corresponding to an effective TE of 100 ms. Simulated magnetization profiles are plotted for white matter relaxation values (T1=832ms,T2=80ms). The excitation pulse, four representative refocusing pulses, and subsequent echo magnetization profiles from the echo train are shown, withou [file MRM-96-214-s001.pdf]

Optimizing Selective RF Pulses for Enhanced Signal Stability in Turbo Spin Echo Using a Differentiable Extended Phase Graph Model

Madison M Augelli[1], Anuj Sharma[1], Mark A Griswold[1,2], and William A Grissom[1,2]

[1]Biomedical Engineering, Case Western Reserve University, Cleveland, OH, USA

[2]Department of Radiology, Case Western Reserve University, Cleveland, OH, USA

February 2, 2026

Address correspondence to:

William A Grissom, Ph.D.

BRB 333

2109 Adelbert Road

Cleveland, OH 44106

wag57@case.edu

This work was supported by NIH grants R01 CA 281043, R01 EB 019437, and T32 EB 007509, NSF GRFP, and Siemens Healthineers.

Contents

1 Truncated Optimized RF Pulses (Fig S1) 3

2 Integrated B_1 and B_1 RMS of Optimized RF Pulses (Fig S2) 4

3 SAR-Matched SLR Pulses: Simulation Results (Fig S3) 5

4 Dependence of RF Pulse Solutions on Echo Train Length (Fig S4) 6

5 Optimized RF Pulses with Constant Signal: Simulation Results (Fig S5) 7

6 Optimized RF Pulses with Constant Signal: Phantom Results (Fig S6) 8

1 Truncated Optimized RF Pulses (Fig S1)

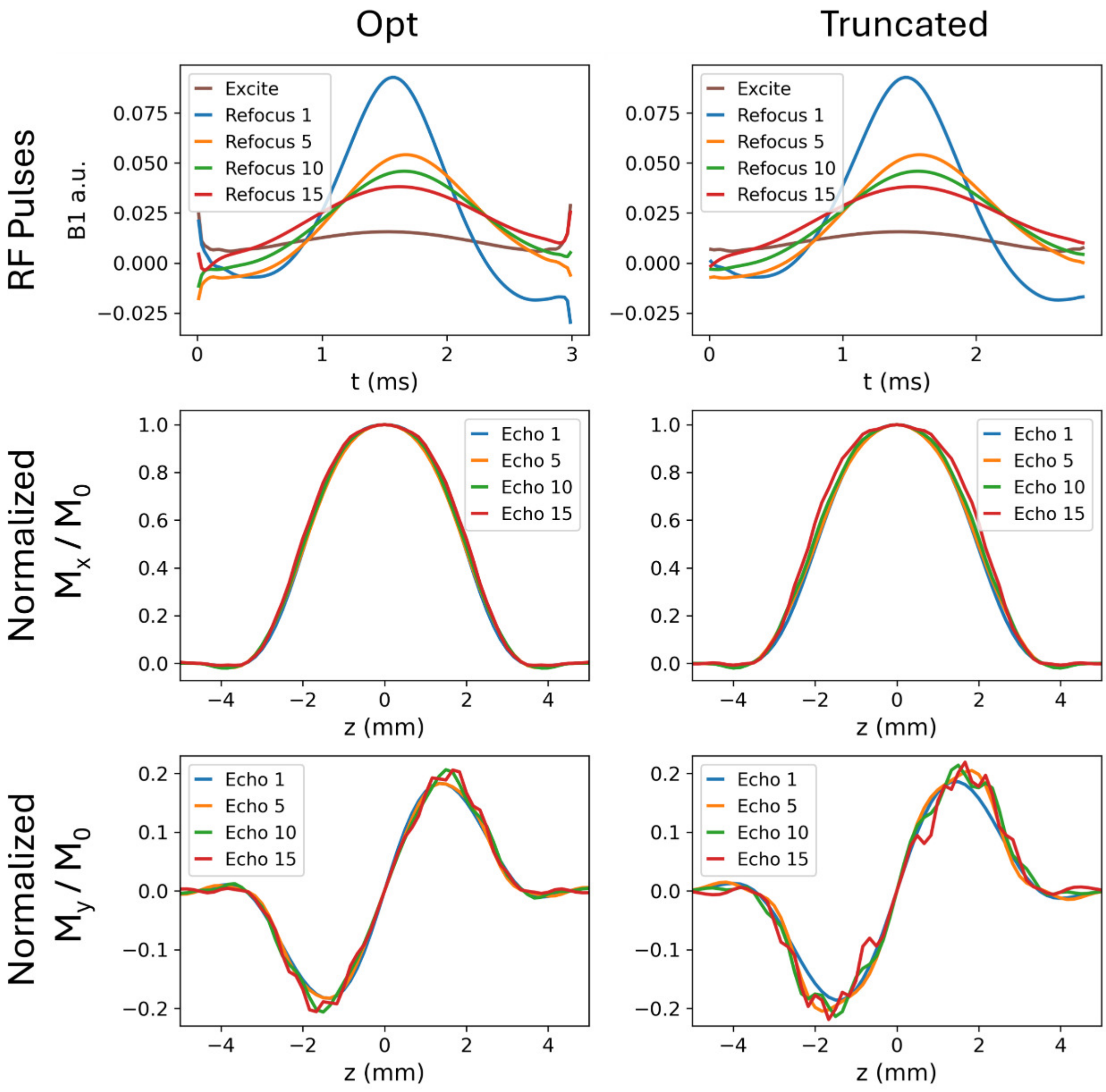

Supporting Information Figure S1: Optimized RF pulses were truncated by removing four time points on both sides to eliminate peaks. The excitation pulse and four refocusing pulses are shown. Normalized M_x and M_y components at the four corresponding echoes are also shown for white matter relaxation values (T_1 = 832 ms, T_2 = 80 ms) to visually assess similarity in shape between echoes.

2 Integrated B_1 and B_1 RMS of Optimized RF Pulses (Fig S2)

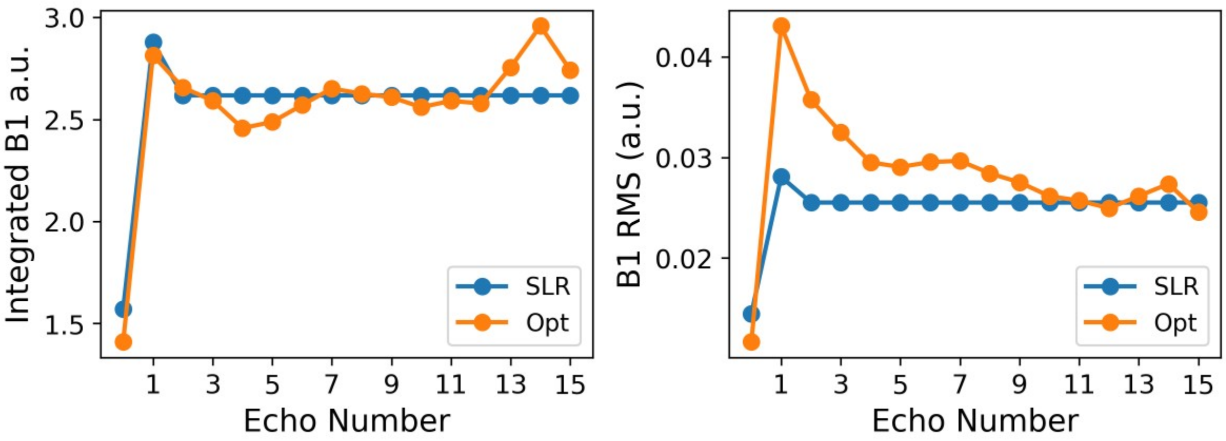

Supporting Information Figure S2: Comparison of the excitation (echo number = 0) and refocusing RF pulse integrated B_1 (left) and B_1 RMS (right) for the SLR and optimized pulses.

3 SAR-Matched SLR Pulses: Simulation Results (Fig S3)

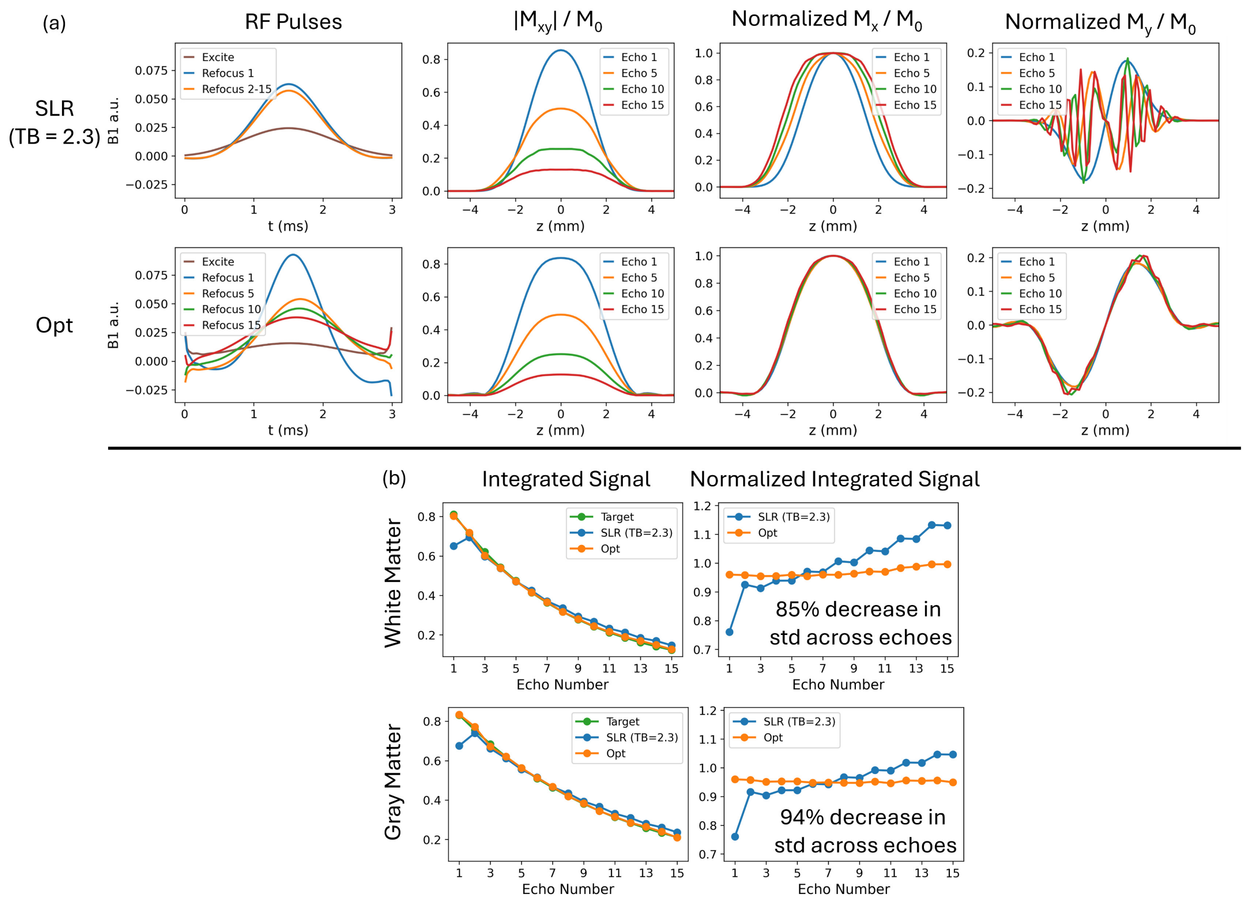

Supporting Information Figure S3: (a) SLR RF pulses with time-bandwidth product 2.3 to match SAR of optimized RF pulses. Simulated magnetization profiles are plotted for white matter relaxation values (T_1 = 832 ms, T_2 = 80 ms). The excitation pulse, four representative refocusing pulses, and subsequent echo magnetization profiles from the echo train are shown, without and with normalization to their peak amplitude. (b) Slice-integrated signals across echoes for white and gray matter, without (left) and with (right) normalization to their peak amplitude.

4 Dependence of RF Pulse Solutions on Echo Train Length (Fig S4)

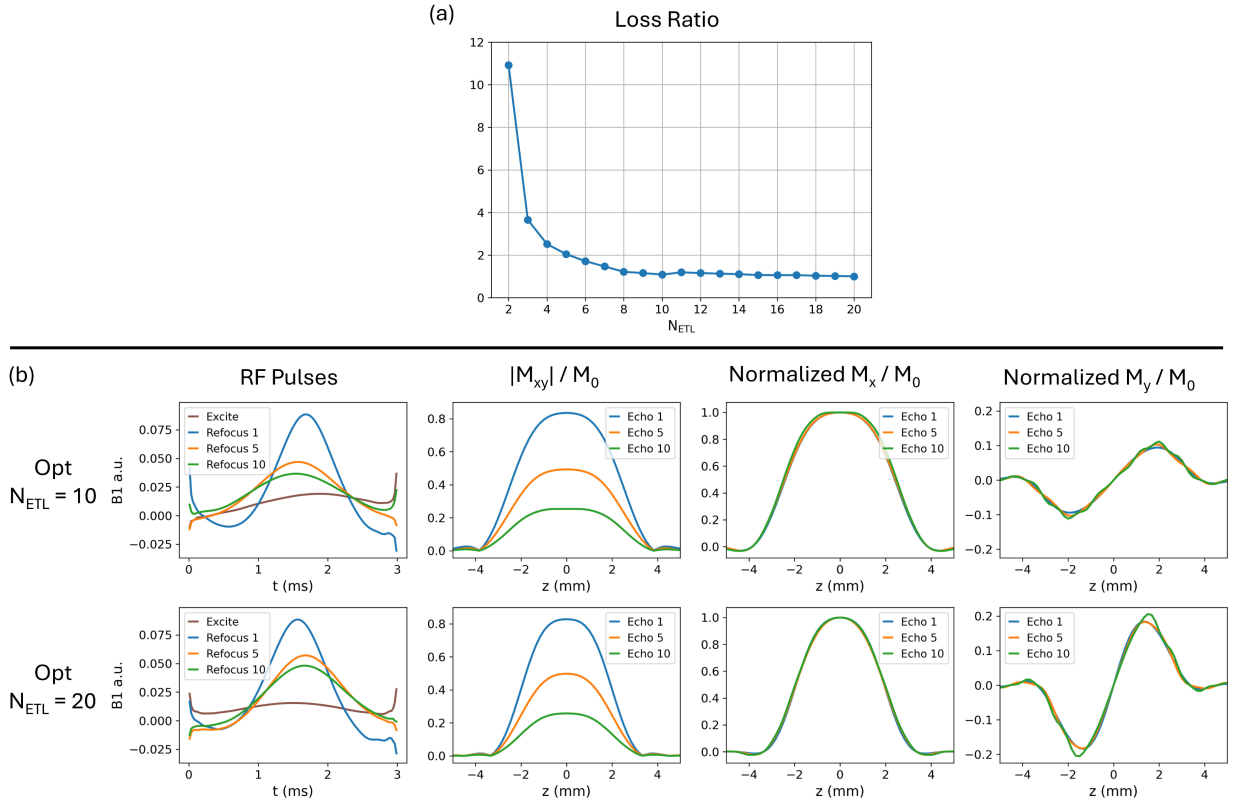

Supporting Information Figure S4: Dependence of RF pulse solutions on echo train length (N_{ETL}). (a) Ratios of design loss (Equation 1) versus N_{ETL} when pulses designed for $N_{\mathrm{ETL}} = 20$ are used for shorter train lengths. Each ratio was calculated as the loss incurred using the twenty-echo pulses at each shorter N_{ETL}, divided by the loss for pulses directly optimized for each N_{ETL}. (b) Comparison of the excitation and four refocusing RF pulse shapes designed for $N_{\mathrm{ETL}} = 10$ (top) versus $N_{\mathrm{ETL}} = 20$ (bottom), and their subsequent magnetization profiles in white matter.

5 Optimized RF Pulses with Constant Signal: Simulation Results (Fig S5)

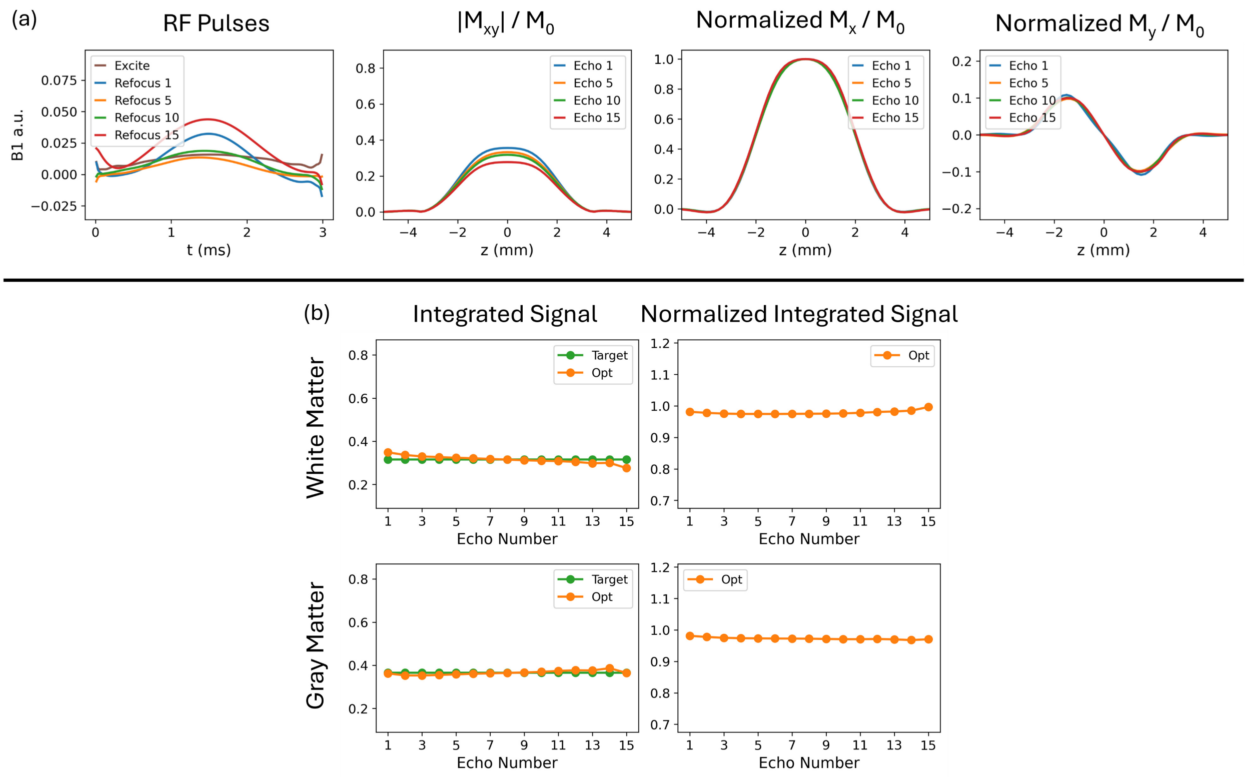

Supporting Information Figure S5: (a) Optimized RF pulses designed to maintain a constant signal equal to the target signal at the ninth echo, corresponding to an effective TE of 100 ms. Simulated magnetization profiles are plotted for white matter relaxation values (T_1 = 832 ms, T_2 = 80 ms). The excitation pulse, four representative refocusing pulses, and subsequent echo magnetization profiles from the echo train are shown, without and with normalization to their peak amplitude. (b) Slice-integrated signals across echoes for white and gray matter, without (left) and with (right) normalization to their peak amplitude.

6 Optimized RF Pulses with Constant Signal: Phantom Results (Fig S6)

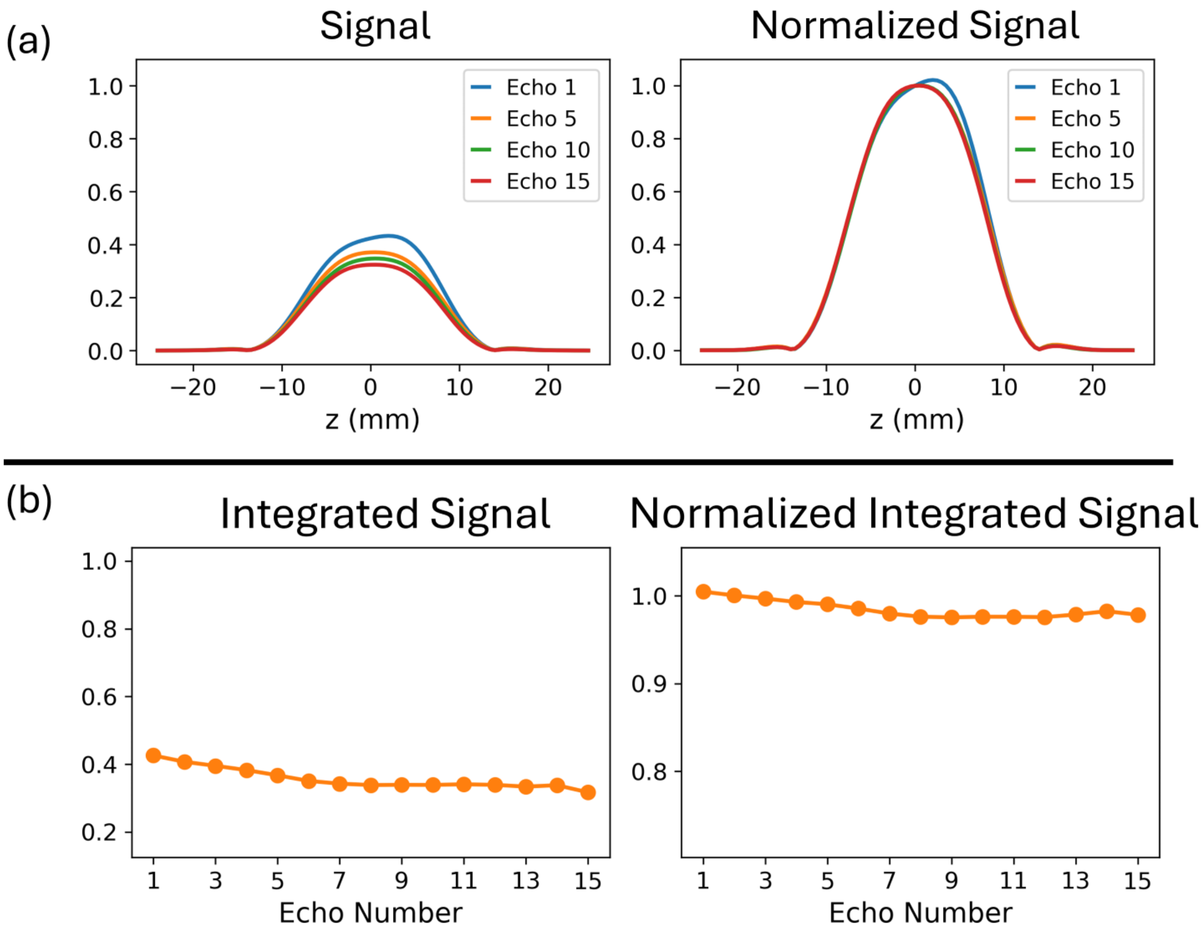

Supporting Information Figure S6: (a) Measured slice profiles from a TSE sequence in a homogeneous phantom using RF pulses optimized to maintain a constant signal equal to that expected at the ninth echo. Four representative echoes are shown (left). To visually compare similarity, each echo was also scaled based on its center value (right). (b) The integrated signal across the slice at each echo (left) and normalized integrated signal (right), calculated by dividing by the echo amplitude, is plotted for the optimized pulses.
